# Updated estimate of the duration of the meningo-encephalitic stage in gambiense human African trypanosomiasis

**DOI:** 10.1186/s13104-015-1244-3

**Published:** 2015-07-04

**Authors:** Francesco Checchi, Sebastian Funk, Daniel Chandramohan, Daniel T Haydon, François Chappuis

**Affiliations:** Faculty of Infectious and Tropical Diseases, London School of Hygiene & Tropical Medicine, Keppel Street, London, WC1E 7HT UK; Centre for the Mathematical Modelling of Infectious Diseases, London School of Hygiene & Tropical Medicine, Keppel Street, London, WC1E 7HT UK; College of Medical, Veterinary and Life Sciences, University of Glasgow, University Avenue, Glasgow, G12 8QQ UK; Operational Centre Geneva, Médecins Sans Frontières, 78 Rue de Lausanne, 1202 Geneva, Switzerland; Division of International and Humanitarian Medicine, Geneva University Hospitals and University of Geneva, rue Gabrielle-Perret-Gentil 4, 1211 Geneva, Switzerland

**Keywords:** Sleeping sickness, Trypanosoma brucei gambiense, Mathematical model, Endemic equilibrium, Screening coverage, Diagnostic algorithm, Duration of infection

## Abstract

**Background:**

The duration of the stages of HAT is an important factor in epidemiological studies and intervention planning. Previously, we published estimates of the duration of the haemo-lymphatic stage 1 and meningo-encephalitic stage 2 of the gambiense form of human African trypanosomiasis (HAT), in the absence of treatment. Here we revise the estimate of stage 2 duration, computed based on data from Uganda and South Sudan, by adjusting observed infection prevalence for incomplete case detection coverage and diagnostic inaccuracy.

**Findings:**

The revised best estimate for the mean duration of stage 2 is 252 days (95% CI 171–399), about half of our initial best estimate, giving a total mean duration of untreated gambiense HAT infection of approximately 2 years and 2 months.

**Conclusions:**

Our new estimate provides improved information on the transmission dynamics of this neglected tropical disease in Uganda and South Sudan. We stress that there remains considerable variability around the estimated mean values, and that one must be cautious in applying these results to other foci.

## Findings

Human African trypanosomiasis (HAT, sleeping sickness) is a neglected tropical disease that progresses from the haemo-lymphatic (early) stage 1 to the ultimately fatal, meningo-encephalitic (late) stage 2 [1]. In a previous paper [2], we reported on the duration of both stages of infection with the Trypanosoma brucei *gambiense* form of HAT, which we estimated at 526 (95% CI 357–833) and 500 (95% CI 345–769) days, respectively. The stage 1 duration estimate was based on survival analysis of untreated stage 1 serological suspects from Uganda and South Sudan foci where Médecins Sans Frontières implemented control programmes: We concluded that the rate $r_1$ of progression to stage 2 was constant, leading to an exponential distribution of stage 1 duration. We then derived the stage 2 duration indirectly based on the stage 1 to stage 2 ratio as per the following equality, resultant from the assumption of endemic equilibrium:

$$\begin{aligned} r_2 = r_1\frac{S_1}{S_2}, \end{aligned}$$

where $r_2$ is the rate of removal from stage 2 (or mortality rate as death appears to be the only possible outcome of untreated stage 2 [3]), also assumed to be constant. In order to quantify the $S_1$ to $S_2$ ratio in typical prevalence conditions, in our original study we then averaged findings from cross-sectional, active case detection (mass screening) sessions performed in villages in Uganda and Southern Sudan, only retaining the analysis sessions during which >70% of the population had presented for testing. Here we present a revised estimate of stage 2 duration, derived by computing the $S_1$ to $S_2$ ratio based on a larger number of screening sessions, without any screening coverage exclusion criteria; and by adjusting observed prevalence data for incomplete screening coverage and diagnostic inaccuracy.

### Methods

Data on mass screening sessions were available from HAT control projects implemented by Médecins Sans Frontières in Adjumani (Uganda, 1991–1996), Arua-Yumbe (Uganda, 1995–2002), and Kiri (South Sudan, 2000–2005), and furnished values of $S_1$ and $S_2$ as well as other analysis parameters. In a recent paper [4], we developed a mathematical modelling method to adjust the $S_1$ and $S_2$ prevalence observed in these sessions so as to account for (1) incomplete screening coverage, by estimating the relative probability of attending screening sessions among cases compared to non-cases; and (2) incomplete sensitivity, specificity and staging accuracy of diagnostic algorithms, which can result in a combination of false negatives, false positives and stage-misclassified cases among persons screened; the diagnostic algorithms used [4] and derived accuracy parameters [5] are described elsewhere. Note that “screening coverage” here refers to the proportion of the population presenting for testing, and is therefore unrelated to how well the screening tests perform.

For this revised analysis, we included screening sessions that yielded at least one case of HAT and were the first to be conducted in the respective villages, i.e., in epidemiological systems as yet unmodified by control (thereby excluding screening sessions done when there was also passive surveillance ongoing), where therefore the $S_1$–$S_2$ ratio tends to reflect natural progression of the disease (ignoring natural mortality, which will occur at a much smaller rate than $r_2$). We took the best estimates of true $S_1$ and $S_2$ prevalence estimated in [4] for each of these screening sessions and summed them across each of the above MSF projects, so as to calculate a mean $S_1$–$S_2$ ratio specific to each HAT focus. We then used this ratio and the estimate of $r_1$ from the original study into the above equation.

This analysis of secondary programmatic data was approved by the Ethics Committee of the London School of Hygiene & Tropical Medicine.

### Results

Overall, 174 active screening sessions were eligible for analysis (Table 1). The ratio of stage 1 and 2 prevalent cases ranged from 1.7 (Kiri) to 2.5 (Arua-Yumbe), for a mean of 2.09. Using the above equation and our previously published estimate of $r_1$ (0.0019 per day, 95% confidence interval 0.0012–0.0028), the revised stage 2 progression (death) rate $r_2$ was 0.0040 per day (95% CI 0.0025–0.0058). Accordingly, the mean duration of stage 2 was 1/0.0040 or 252 days (95% CI 171–399), and the mean total duration of stage 1 and 2 combined was 778 days (95% CI 528–1232), or approximately two years and two months. Similarly, the median stage 2 duration was 175 days (95% CI 119–277), giving a total median gambiense HAT duration of 539 days (95% CI 367–855), or 1 year and 6 months.

**Table 1:** Details of eligible active screening sessions and best estimates of stage 1 and 2 prevalence in the three areas investigated, as well as in all three areas combined (equivalent to treating the three screened populations as one large population)

	Adjumani, Uganda	Arua-Yumbe Uganda	Kiri, South Sudan	Combined
Number of eligible screening sessions	78	64	32	174
Total population targeted for screening	76,864	163,523	11,231	251,618
Village population: median (IQR)	831 (630–1,074)	2,274 (1,824–2,844)	217 (103–297)	1,041 (571–2035)
Estimated prevalent cases in stage 1 (S_1_)	731	298	101	1130
Estimated prevalent cases in stage 2 (S_2_)	363	117	61	541
*S*_1_–*S*_2_ ratio	2.01	2.55	1.66	2.09

*IQR* inter-quartile range.

### Discussion

The revised best estimate is about half of that presented in our initial publication (albeit with overlapping confidence intervals). While the original estimate was derived using only a sub-set of screening sessions, with a cut-off for proportion of the population screened, and was based on an incomplete adjustment for diagnostic inaccuracy of the testing algorithms, the revised estimate includes many more sessions and accounts for diagnostic inaccuracy. Differences between the two may well be explained by including a larger dataset and by avoiding potential bias introduced by the cut-off.

However, the revised estimate is otherwise subject to the same potential sources of bias discussed at length in the original publication [2], which include: (1) bias in the estimate of r_1_, due to high loss to follow-up and possible systematic differences between the serologically suspect patients included in the analysis of stage 1 duration and more typical HAT cases; and (2) bias in the estimate of the* S*_1_+ to* S*_2_ ratio, which relies on assumptions of stable incidence and homogeneity across different HAT foci. We stress that there remains considerable variability around the estimated mean values, and that one must be cautious in applying the results to other foci.

Further analyses are needed, particularly in light of documented differences in the clinical profile and duration of illness of both gambiense and rhodesiense HAT across Sub-Saharan Africa [3, 6, 7]. By applying the model to other foci might, it might be possible to obtain more general conclusions about the progression of the disease and underlying heterogeneities.

## Abbreviations

HAT: human African trypanosomiasis
IQR: inter-quartile range
MSF: Médecins Sans Frontières
*S*_1_: point prevalence of HAT cases in stage 1 of the disease
*S*_2_: point prevalence of HAT cases in stage 2 of the disease
*r*_1_: rate of progression from stage 1 to stage 2
*r*_2_: rate of removal from stage 2 (through death or spontaneous cure).
